# Extragonadal Mixed Germ Cell Tumour of the Right Scapular Region: A Case Report

**DOI:** 10.31729/jnma.7910

**Published:** 2022-12-31

**Authors:** Sushma Gurung, Sabina Karim, Sagun Thapa, Shristi Gautam

**Affiliations:** 1 Department of Paediatric Hematology and Oncology, Bhaktapur Cancer Hospital, Dudhpati, Bhakatapur, Nepal; 2 Department of Paediatric Hematology and Oncology, National Institute of Cancer Research and Hospital, Mohakhali, Dhaka, Bangladesh

**Keywords:** *germ cell tumor*, *immunohistochemistry*, *tumor markers*

## Abstract

Extragonadal germ cell tumours are rare; to the best of our knowledge, a location in the soft tissue of the right scapular region has never been previously reported in the literature. We report a case of a 9-years-old girl who presented with swelling over the right scapular region, treated by a combination of surgery and cisplatin-based chemotherapy. Immunohistochemistry and serum tumour markers concluded it to be an extragonadal mixed germ cell tumour. Our patient had a complete response up to 2 years of follow-up. This case is being reported here due to a very rare site of presentation with a diagnostic dilemma. A multidisciplinary, combining systemic chemotherapy and surgery is the most appropriate treatment strategy for extragonadal germ cell tumours, to ensure both local and systemic control.

## INTRODUCTION

Germ cell tumours (GCTs) account for approximately 2-3% of childhood malignancies under 15 years of age.^1^ They mostly arise in midline sites. They are more common in the ovaries and testes than in extragonadal sites in children older than 2 years, however, 1-5% of all GCTs occur in extragonadal sites.^2^ The most common locations in order of frequency in children are sacrococcygeal (42%), mediastinum (7%), intracranial (6%), and retroperitoneum (4%). Sacrococcygeal teratoma is the most common GCT of childhood accounting for 40% of extragonadal germ cell tumours (EGGCTs).^3^ Extragonadal localisation of germ cell tumours other than midline sites are very rare and to date, there has been no reported case of germ cell tumour arising from the scapular region.

## CASE REPORT

**Figure 1:**
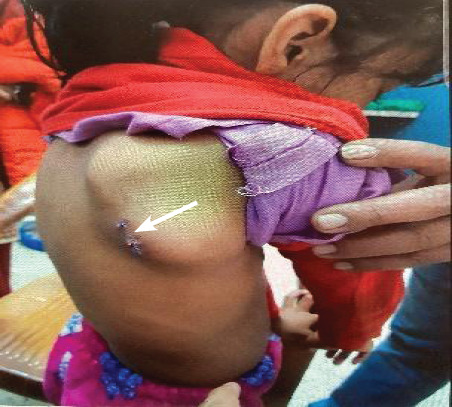
A mass measuring approximately 3 x 4 cm over the right scapular region.

A 9-years-old girl presented with pain and progressive swelling over the right scapular region for a month following trauma with no other significant history. Physical examination revealed a non-tender mass over the right scapula measuring approximately 3x4 cm (Figure 1).

**Figure 2:**
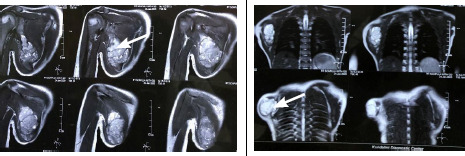
A) An MRI scan showed an ill-defined altered signal intensity lesion measuring 49x35 mm noted at the infraspinatus portion of the body of the scapula, B) heterogeneous enhancement of the lesion on the post-contrast study.

A magnetic resonance imaging (MRI) scan showed an ill-defined altered signal intensity lesion measuring approximately 49x35 mm (hypodense on T1, hyperintense on T2, and STIR) noted at the infraspinatus portion of the body of the scapula. The lesion is causing bony destruction with large soft tissue components involving adjacent muscles, predominantly infraspinatus, pectoralis minor, subscapularis, and teres major. There is a heterogenous enhancement of the lesion on the post-contrast study likely to be neoplasm probably Ewing sarcoma (Figure 2).

A biopsy from the mass was done and morphological features favour immature teratoma. However, the site of the lesion was not compatible with the diagnosis, and immunohistochemistry (IHC) was sent which was consistent with synovial sarcoma. IHC markers showed non-immunoreactive for Spalt-like transcription factor (SALL-4) and cluster determinant 30 (CD30), patchy immunoreactive (1+) for Glypican-3, and immunoreactive (2+) for Cytokeratin(CK) and (4+) for Cluster of differentiation 99 (CD99) and Transducin-like enhancer of split 1(TLE-1) but alfa fetoprotein (AFP) was not stained. Initial levels of AFP were raised (303 ng/dl) and B-human chorionic gonadotrophin (B-HCG) and serum lactate dehydrogenase (LDH) levels were within normal ranges. A computed tomography (CT) scan of the head, thorax, abdomen, and pelvis showed no abnormalities.

**Figure 3:**
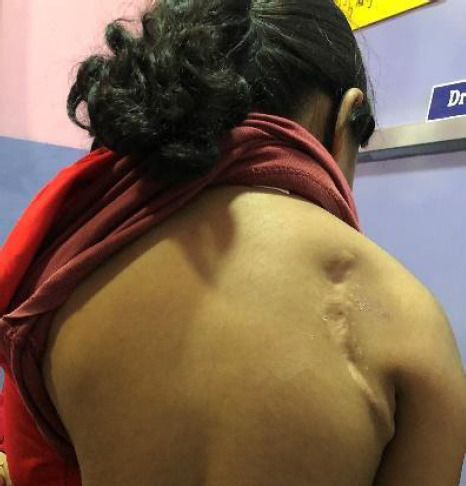
Wide local resection with partial scapulectomy of the mass.

Based on the histopathology report and raised serum AFP with a high suspicion of EGGCT, a repeat IHC was sent. Then, the patient was treated with the first cycle of chemotherapy containing cisplatin, etoposide, and bleomycin (PEB) regimen for extragonadal malignant mixed GCT-stage III. Repeat IHC report was consistent with Germ Cell tumour (non-seminomatous) and showed focal positive for CK, Smooth muscle actin(SMA), desmin and positive for S100, Vimentin, and SALL4. AFP showed weak positivity. An excision specimen with more representative tissue was advised and needs to be examined to confirm the rendered diagnosis. Also, due to poor response to chemotherapy, surgical (Musculoskeletal Oncology) consultation was done and wide local resection with partial scapulectomy was performed (Figure 3).

Final pathology revealed a malignant mixed non-seminomatous germ cell tumor (immature teratoma grade 3 and yolk sac tumour comprises approximately 20% of the tumour examined). Cut margins were free of tumours.

**Figure 4:**
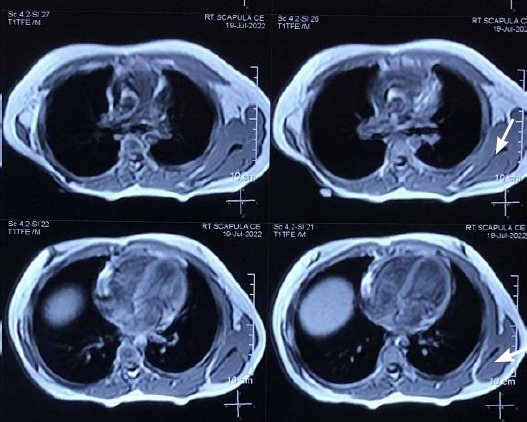
Follow-up MRI at 2 years showed no evidence of recurrence.

The patient was treated with further 3 cycles of the same chemotherapy protocol (PEB regimen). At the end of chemotherapy, serum AFP level was found within normal limits (1.12 ng/dl) and subsequent imaging did not show any pathologic findings (Figure 4).

The patient was advised for regular follow-up with tumour marker and imaging and she had no evidence of tumour recurrence up to 2 years of follow-up.

## DISCUSSION

Primary GCTs of extragonadal origin comprise 1-5% of all germ cell tumours.^2^ They mostly arise in the midline sites, from the brain to the coccyx. Isolated cases have been reported in the bladder, prostate, paratesticular, adnexa, vulva, placenta, pelvis, uterus, kidney, and nasal sinuses.^4^ Herein, we describe what is to the best of our knowledge the first reported case of a mixed germ cell tumour located in the scapular region.

There are multiple theories regarding the development of EGGCTs. The first hypothesis is that EGGCTs are derived from primordial germ cells that fail to complete the normal migration along the urogenital ridge to the gonadal ridges during embryonal development. This may be due to an abnormality in the primordial germ cell itself or in its microenvironment.^5^ The second main hypothesis is that germ cells transformed in the testes undergo reverse migration.^5^ Another theory suggests that EGGCTs develop when germ cells that have regularly spread during embryogenesis into the liver, bone marrow, and brain undergo malignant transformation.^6^ EGGCTs could represent metastasis developed from an undiagnosed or regressed ("burned out") primary GC tumors in the gonads.^7^

A biopsy is required for definitive diagnosis of EGGCTs. The diagnosis of teratoma generally does not require IHC, but it could be needed in the case of suspected immature components.^8^ In our patient, the pathological diagnosis was immature teratoma, so IHC was sent. Also, the most commonly increased serum tumor markers (STMs) in EGGCTs patient include AFP, B-HCG and LDH. Our patient had initial AFP level raised. Serum AFP is almost always increased in pure Yolk Sac Tumor (YST) or containing YST-mixed GCT patients.^8^ Therefore, though the initial IHC report was suggestive of synovial sarcoma, considering histopathology and elevated AFP, a repeat IHC was sent which was consistent with non-seminomatous germ cell tumour.

A multimodality approach with chemotherapy followed by surgery for residual masses is considered the standard of treatment for EGGCTs. The introduction of cisplatin-based chemotherapy as in gonadal counterpart has dramatically improved the prognosis.^8^ Because an extragonadal GCT may be curable with cisplatin-based chemotherapy, many recommend that the diagnostic evaluation in such cases should include measurement of the STMs and immunohistochemical assessment of the biopsy. Our patient received the first cycle of chemotherapy (PEB regimen). As there was no response clinically and with STMs, wide local excision with partial scapulectomy was performed after the first cycle of chemotherapy. In the case of localized EGGCTs, patients can really benefit from radical surgery and subsequent chemotherapy also.^9^ Final pathology of our patient revealed immature teratoma with approximately 20% of the yolk sac component. Thus, the presence of a residual tumour is often due to unresponsiveness of teratoma components in EGGCTs mass.^10^ Indeed, surgical excision demonstrated advantages in order of disease free-survival, when complete excision can be performed.

This is a rare case of extragonadal germ cell tumour of the scapular region. Regardless of the location, the therapeutic approach of extragonadal GCTs should be multidisciplinary, combining systemic chemotherapy and surgery. This study revealed the importance of tumour markers, especially in doubtful cases along with extended IHC panels in the diagnosis of EGGCTs with immature components.
